# Effects of Flooding and Endogenous Hormone on the Formation of Knee Roots in *Taxodium ascendens*

**DOI:** 10.3389/fpls.2022.803619

**Published:** 2022-02-03

**Authors:** Zhuangzhuang Qian, Lin Wu, Luozhong Tang

**Affiliations:** Co-innovation Center for Sustainable Forestry in Southern China, College of Forestry, Nanjing Forestry University, Nanjing, China

**Keywords:** anatomic structure, ethylene, flooding, formation mechanism, knee root, *Taxodium ascendens*

## Abstract

*Taxodium ascendens* is a typical tree species with high flood tolerance, and it can generate knee roots in the wetlands. This study investigated the number and size of knee roots and the soil flooding conditions. Furthermore, we also measured physiology, biochemical responses, and the anatomical structure of knee roots and underground roots at different developmental stages. This study aimed to understand the adaptation mechanism of *T. ascendens* to flooding stress and the formation mechanism of the knee roots. The results showed that the formation of knee roots was significantly affected by the soil water table (*P* < 0.05). The middle water table was more conducive to the formation of knee roots. In the middle water table, the 1-aminocyclopropane-1-carboxylic acid (ACC) content and ACC synthase activity were significantly lower in the knee roots than in the underground roots. The knee roots at the young-aged stage showed the highest ACC oxidase activity among the development stages of the knee roots. The ethylene release rate was significantly higher in the knee roots than in the underground roots (*P* < 0.05). Indole-3-acetic acid (IAA) content first increased, then decreased with knee root development. The periderm cells at the apex of the knee roots were dead and had many intercellular spaces, which was beneficial for the growth of *T. ascendens*. In conclusion, the middle water table induced the ethylene and IAA production, which promoted the formation of knee roots, which improved roots ventilation and flooding tolerance of *T. ascendens*. The results obtained can provide information about mechanisms of knee roots formation and provide scientific evidence for the afforestation and management under wetland conditions.

## Introduction

Nowadays, flood damage is receiving a lot of attention, as flooding phenomena tend to be closely associated with global climate changes (Hirabayashi et al., 2013; Zhou et al., 2020). Flooding is a major abiotic stress that determines species distribution, growth, and yield (Herzog et al., 2016). In China, about 6.6 × 10^5^ km^2^ land is waterlogged, accounting for 6.6% of the total land area; in 2020, 16 provinces and 30.20 million people were adversely affected by floods. Uneven precipitation and poor drainage result in frequent waterlogging, seriously constrain crop growth and productivity (Kuai et al., 2015; Xiong et al., 2019). Therefore, the selection of a suitable farmland shelter forest is essential. Flooding stress can impede the gas exchange between soil and atmosphere, restricting oxygen diffusion in plant tissues. Since oxygen is critical for mitochondrial respiration, this process cannot be maintained under flood conditions, which seriously constrains plant growth (Voesenek and Baileyserres, 2013).

Flood-tolerant plant species have a series of adaptive mechanisms, such as morphological changes, physiological and biochemical reactions, that can protect the plants from flooding stress (Khan et al., 2020). For example, the structures of some Chenopodiaceae roots change to the herring-bone shape to consume less oxygen and avoid being damaged by toxins (Bouma et al., 2001). The red mangrove (*Rhizophora mangle*) in coastal intertidal zones formed stilt-like roots for gas exchange and prevention of oxygen loss during flooding stress (Lance and Alison, 2010; Mendez-Alonzo et al., 2015). *Avicennia marina* can develop finger-like pneumatophores to obtain oxygen for its underground root system under hypoxic conditions (Purnobasuki et al., 2017). *Eucalyptus camaldulensis* can produce numerous white adventitious roots that float on the water surface to obtain more oxygen (Sena Gomes and Kozlowski, 1980).

*Taxodium ascendens* is a flood-tolerant species widely distributed in the subtropical riversides, drawdown areas of reservoirs, and ponds in China (Yi et al., 2008; Li et al., 2010). *T. ascendens* develops knee roots to increase water tolerance under flooding conditions; it is widely cultivated in farmland shelter forests and avenue plantations (Tang et al., 2008; Du et al., 2010). Knee roots are morphological changes of underground roots, which look like a cone bulging from the ground. They result from woody bulges within the secondary root cambium, which increases the thickness of the upper surfaces of the underground roots; the irregular growth of the upper and below parts of roots leads to the special shape (Tang et al., 2008). Sakio and Yamamoto (2002) have reported that only specific flood-tolerant plant species like *T. ascendens*, *Taxodium distichum*, and *Glyptostrobus pensilis* develop knee roots.

Although various studies have reported the effects of waterlogging on the metabolic and physiochemical characteristics of different trees, most of the studies were conducted in greenhouse conditions and focused on small tree seedlings (Conner et al., 1997; Andrson and Pezeshki, 1999; Li et al., 2010; Wang et al., 2016). Few studies were performed using adult trees under field conditions (Sou et al., 2019). Therefore, the adaptation mechanism of *T. ascendens* roots to waterlogging stress is unclear because the knee roots are only generated in adult trees in the field condition. Tang et al. (2008) found that the middle water table was beneficial for the formation of the knee roots. Yamamoto (1992) reported the flooding stress stimulated ethylene production by stem bark and the apex of knee roots and hypothesized the ethylene and indole-3-acetic acid (IAA) jointly contributed to knee root production. Ethylene can promote plant growth, seed germination, as well as enhance anoxia resistance (Deikman, 1997). Especially, ethylene, as an internal gaseous hormone, is used by plants to sense shifts from aerial to aquatic environments and induce changes in plant morphology and anatomy (Visser and Voesenek, 2004; Jackson, 2007). Sundberg et al. (1991) indicated that endogenous IAA is a critical regulator in the differentiation of the vascular strands, and significantly affects cambial activity and development. 1-Aminocyclopropane-1-carboxylic acid synthetase (ACS) and 1-Aminocyclopropane-1-carboxylic acid oxidase (ACO) are essential enzymes that participate in ethylene production (Dong et al., 1992; Vall-llaura et al., 2020). Application of ACC combined with IAA on the incision of stems of *T. distichum* seedlings was demonstrated to strongly accelerate the formation of abnormal tracheids (Yamamoto, 1992). Tavares et al. (2018) also suggested that the sensitivity of ethylene and the balance between ethylene and IAA result in forming aerenchyma.

Therefore, we hypothesized that the flooding condition affected the knee roots formation; the synthesis of endogenous hormones can promote the development of knee roots and contribute to the adoption of *T. ascendens* under flooding conditions. We aimed to explore the suitable water table for the knee roots formation and the growth of *T. ascendens*; to compare endogenous hormone levels between different development stages of knee roots and conventional underground roots; to understand the adaptation mechanism of *T. ascendens* under flooding conditions and form a basis for afforestation management under wetland conditions.

## Materials and Methods

### Research Site

The study was performed in a 28-year-old *Taxodium ascendens* forest plantation (approximately 500 hectares) with a mean diameter at breast height (DBH) of 25.75 cm and an average tree height of 14.33 m. The plantation was cultivated in a wetland at the Zhaoguan Forest Farm (N32°31′41″, E119°30′35″), Jiangdu County, Jiangsu Province, China. The plant spacing was 1.5 m × 4 m. The soil type was paddy soil with poor air permeability. The plant materials were selected with permission from the Zhaoguan Forest Farm; no ethics approvals were required.

### Experimental Design, Analysis of the Soil Water Table and Knee Root Number and Size

**FIGURE 1:**
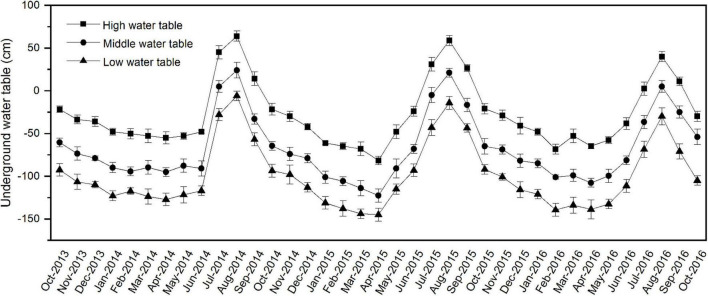
Monthly variation of underground water table during the experimental period from October 2013 to October 2016 in different study sites. The error bars represent standard deviation (SD) of the underground water table.

A complete-randomized block design was used in this experiment. From October 2013 to October 2016, the soil water table at each site was measured every month using polyethylene pipes (length, 2 m; inner diameter, 11 cm). The pipes were drilled with 4 mm diameter holes (the spacing between each hole was 5 cm), then vertically buried in the soil at each site. According to the underground water table (Figure 1), three experimental sites were chosen based on the soil water table. High water table site (the flooding period was more than 3 months from June to September, maximum submergence depth was about 0.6 m, the annual average water table was about −0.3 m). Middle water table site (the flooding period was 1–2 months from July to August, maximum submergence depth was about 0.2 m, the annual average water table was about −0.7 m). Low water table site (no flooding status, the average yearly water table was about −1.0 m). A total of 21 plots (7 sampling plots at each water-table site) were established. Each plot was 96 m^2^ (8 × 12 m).

The tree height, DBH, and number of knee roots were measured in October 2015. The knee roots’ height and average diameter were also measured at half the height of the knee roots. Due to the irregular surface of the knee roots, we carefully wrapped the knee roots with tape (width, 1.62 cm). The surface area was calculated according to the length and width of the tape (with no overlap in the wrapping process). The survey of underground roots with knee roots and without knee roots was randomly conducted in 15 plots (1 m × 1 m) within a range of 2 m from the trunk of the *T. ascendens*. The underground roots (depth, 0–50 cm) were excavated, cleaned, dried, and weighed.

### Sampling of Knee Roots and Assay of Physiological Indicators

**FIGURE 2:**
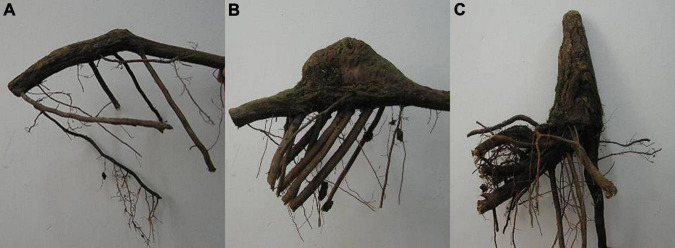
Photographs of knee roots of young-aged stage **(A)**, middle-aged stage **(B)**, and old-aged stage **(C)**. The knee roots at the young-aged stage were less than 5 cm in height and less than 5 years of age. The knee roots at the middle-aged stage were 5–10 cm in height and 5–10 years of age. The knee roots at the old-aged stage were more than 10 cm in height and more than 10 years of age.

The sampling was also conducted in August 2016. Since the best growth condition and the highest knee roots numbers were observed in the middle water table, the middle water table sites were chosen to investigate the development stages of the knee roots. The knee roots were divided into three stages based on size and age: young-aged stage (KY, Figure 2A), middle-aged stage (KM, Figure 2B), and old-aged stage (KO, Figure 2C). The knee roots at the young-aged stage were less than 5 cm in height and less than 5 years of age. The knee roots at the middle-aged stage were 5–10 cm in height and 5–10 years of age. The knee roots at the old-aged stage were more than 10 cm in height and more than 10 years of age. The age of the knee roots was determined using the annual rings in the transverse section. The knee root samples were divided into the swollen (upside) and the non-swollen part (underside). Underground roots approximately 10 mm in diameter were collected as the control. After removing the bark, the fresh root tissues, including cambium and phloem, were collected for physiological and biochemical analysis.

### Ethylene Release Rate of the Roots

According to Kurepin et al. (2010), the ethylene release rate was determined with minor revisions. Fresh root tissues (0.6 g) were sealed in a closed bottle, incubated for 4 h at 30°C. Then, an airtight syringe injected 0.5 mL of gas samples into a gas chromatograph (Agilent 7890A; Agilent Technologies Inc. United States). The gas chromatograph was equipped with a flame ionization detector, electron capture detector and HP-PLOT Al_2_O_3_ S column (50.0 m × 0.53 mm × 0.15 μm, Agilent Technologies Inc. United States). The initial temperature of the column was set as 100°C for 10 min. Then the column was heated to 180°C for 90 min. The carrier gas (nitrogen) flow rate was 30 mL min^–1^. The flow rate of hydrogen was 35 mL min^–1^. The airflow rate was 450 mL min^–1^. The external standard method was used to calculate the ethylene concentration of the samples.

### 1-Aminocyclopropane-1-Carboxylic Acid Content

The ACC content was determined according to Hoffman et al. (1983). The root tissue samples were cut into small pieces and mixed completely. Then, the samples (1.0 g) were homogenized in 8 mL of ethanol (95%) and centrifuged (8,000 × *g*) at 4°C for 15 min. The supernatant was transferred to a plastic bottle, 6 mL of ethanol (80%) was added to the residue and shaken for 30 min. After centrifugation, the supernatant was transferred to the previous bottle and dried in a water bath at 40°C. The dried residue was dissolved with 5 mL of distilled water, then centrifuged (8,000 × *g*) at 4°C for 10 min. The supernatant was used as the ACC fluid; 1 mL of the fluid and 40 μL of HgCl_2_ (25 mmol⋅L^–1^) were added into test tubes (20 mL) closed with rubber stoppers. Then, a syringe injected 1 mL of NaOCI–NaOH (v:v = 2:1) into the test tubes. After mixing up, gas samples (0.5 mL) were collected from the tubes to determine the ethylene concentration *via* gas chromatography. The ACC concentration of fresh root samples was calculated using the following formula:


ACC⁢content⁢(nmol⋅g⁢FW-1)=C×VL×VR×V1×V2×W×22.4

where C is the ethylene concentration measured using gas chromatography (nL⋅L^–1^); V_*L*_ is the volume of sample bottles without solution (mL); V is the volume of the extracting solution (mL); R is the transfer coefficient from ACC to ethylene; V_1_ is the volume of the extracting solution used for measurement (mL); V_2_ is the gas sample volume for gas chromatography (mL); W is the fresh weight of the root sample (g); 22.4 is 1 molar gas constant under normal atmospheric conditions (L⋅mol^–1^).

### 1-Aminocyclopropane-1-Carboxylic Acid Synthetase Activity

The ACS activity was determined according to Mehta et al. (1988). Briefly, 1 g of root tissues were ground in a mortar with the extraction buffer (2 mL) and centrifuged at 10,000 × *g* for 20 min at 4°C. The extraction buffer solution contained 400 mmol⋅L^–1^ potassium phosphate buffer solution (pH 8.5), 1 mmol⋅L^–1^ ethylene diamine tetraacetic acid, 0.5% (v:v) β-mercaptoethanol, and 10 μmol⋅L^–1^ pyridoxal phosphate (PLP). The supernatant was used to determine ACC enzyme activity; 0.4 mL of the enzyme extract and 1.6 mL of the buffer solution (containing 50 μmol⋅L^–1^ S-adenosylmethionine (SAM), 10 μmol⋅L^–1^ PLP, and 50 mmol⋅L^–1^ Hepes-KOH, pH 8.5) were added to the test tubes. After incubation (32°C, 1 h), 0.1 mL of mercuric chloride (500 mmol⋅L^–1^) stopped the reaction. The test tubes were closed, incubated in ice water for 5 min. 0.2 mL of 5% NaOCI-NaOH (v:v = 2:1) was injected into the test tubes. After shaking, a 0.5 mL gas sample was collected to determine the ethylene content using gas chromatography.

### 1-Aminocyclopropane-1-Carboxylic Acid Oxidase Activity

According to Dong et al. (1992), the ACO activity was determined. 0.5 g of root tissues were ground in a mortar with the extraction buffer (2 mL) and centrifuged at 12,000 × *g* for 10 min at 4°C. The extraction buffer solution contained 100 mmol⋅L^–1^ Tris-HCl (pH 7.5), 10% (v:v) glycerin, 30 mmol⋅L^–1^ sodium ascorbate, 5% (v:v) polyvinylpyrrolidone, 0.1 mmol⋅L^–1^ FeSO_4_, and 5 mmol⋅L^–1^ DTT. The supernatant was used to determine ACO activity; 0.2 mL of the enzyme extract and 1.8 mL of the buffer solution (containing 100 mmol⋅L^–1^ Tris-HCl (pH 7.5), 10% (v:v) glycerin, 30 mmol⋅L^–1^ sodium ascorbate, 30 mmol⋅L^–1^ NaHCO_3_, 1.0 mmol⋅L^–1^ ACC, and 0.1 mmol⋅L^–1^ FeSO_4_) were added to test tubes (20 mL) closed with rubber stoppers. After incubation (35°C, 20 min), a 0.5 mL gas sample was collected to determine the ethylene content using gas chromatography.

### Indole-3-Acetic Acid Content

The IAA content was determined according to Chen and Zhao (2008) and Yuan et al. (2008). After homogenization, 1 g of root tissues were extracted in precooled 80% methanol and butylated hydroxytoluene (1 mmol⋅L^–1^) overnight at 4°C. After centrifugation, the supernatant was passed through a C18 Sep-Pak Cartridge (Waters Corp., Milford, United States), and dried in a rotary evaporator (RE-2000A, China). The residue was collected in 0.8 ml mobile phase (consisting of 23% (v:v) methanol and 23% (v:v) acetonitrile in double distilled water supplemented with 0.1% (v:v) phosphoric acid), filtered through a 0.25 mm filter for High Performance Liquid Chromatography (HPLC) analysis. The IAA content was determined using the external standard method with a Waters 2695 Alliance HPLC (Waters Corp.) equipped with a Symmery C18 column (Waters Corp.) (4.6 × 250 mm, 5 μm).

### Anatomical Analysis of Knee Roots and Underground Roots

**FIGURE 3:**
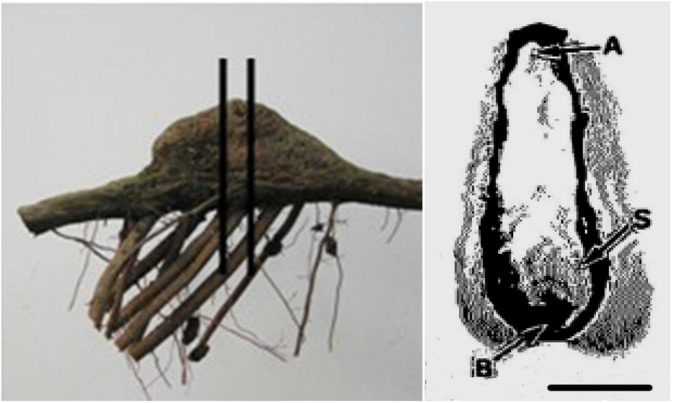
Photographs of a root segment with a knee root **(left)** and transverse section of a knee root **(right)**. Vertical bars indicate the portion with the transverse section of the knee root. Letters indicate positions of samples for microscopic observations. The horizontal bar equals 2 cm. A, apex; B, base; S, side.

The thickness of the rhytidome, size of the secondary phloem, phloem parenchyma and earlywood tracheids from the apex of underground roots and knee roots at different development stages were determined according to Yamamoto (1992). The anatomical analysis was performed according to Yamamoto (1992) and Zhang et al. (2013). Three samples containing bark and currently produced xylem from the apex of knee roots at the middle-aged stage and mid-sized underground roots were obtained (Figure 3). Small pieces (10 × 10 × 10 mm) of these root materials were fixed in FAA solution (formalin: acetic acid: ethanol: water, 5:5:60:30, v:v) for 24 h, rinsed in water, dehydrated in ethanol, and sealed in a paraffin block. The samples were transversely sectioned (10 μm) with a slide microtome (Leica RM2125RT), dewaxed, stained with safranin-fast green solution, and oven-dried at 40°C. The anatomical structure of the samples was observed under a light microscope (Olympus, Japan), the pictures were taken and processed using an image analysis software (DT2000, China).

### Statistical Analysis

All data were subjected to a one-way analysis of variance by using IBM SPSS Statistics 19.0 (IBM Corp., Armonk, NY, United States). Pearson’s correlation analysis was performed using IBM SPSS Statistics 19.0 (IBM Corp). The data were presented as mean ± standard deviation (M ± SD) values, and the means of 3 replicates were evaluated using Duncan’s test at a significance level of 0.05.

## Results

### Relationships Between the Morphological Characteristics of Knee Roots and Underground Water Table

The results showed that the water table significantly affected the formation and distribution of knee roots (*P* < 0.05). The knee root density in the middle water table was significantly higher than that in the high water table and low water table (*P* < 0.05). The height and diameter of the knee roots were also observed to be significantly higher in the middle water table site (*P* < 0.05) (Table 1).

**TABLE 1:** Number and size of knee roots of *Taxodium ascendens* at different water table sites.

Site	Annual water table (m)	Flooding period (month year^–1^)	Knee root density (root m^–2^)	Knee root height (cm)	Knee root diameter (cm)
High water table	−0.3	> 3	0.66 ± 0.02 b	6.65 ± 1.36 b	3.73 ± 0.12 b
Middle water table	−0.7	1–2	1.61 ± 0.64 a	7.64 ± 1.42 a	4.11 ± 0.51 a
Low water table	−1.0	0	0.65 ± 0.02 b	6.72 ± 1.60 b	3.70 ± 0.25 b

*Data represent mean ± SD values of seven replication sites.*

*Values followed by the same letter are not significantly different at P < 0.05, according to Duncan’s multiple range tests. The height of the knee roots refers to the height from the ground to the apex of the knee roots. The knee root diameter refers to the average diameter at half the height of the knee roots.*

### Effects of the Knee Roots on the Growth of *Taxodium ascendens*

**FIGURE 4:**
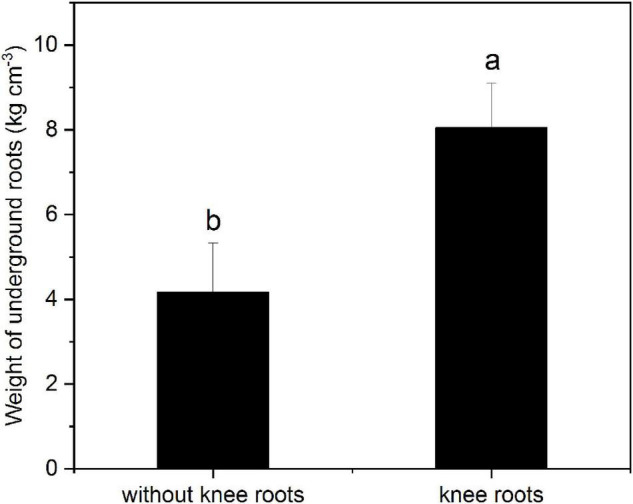
Weight of underground roots with or without knee roots. Values followed by the same letter(s) are not significantly different at *P* < 0.05, according to Duncan’s multiple range tests. Error bars are SD of the mean; *n* = 3.

As shown in Figure 4, the weight of underground roots with knee roots was significantly higher than that without knee roots (*P* < 0.05). Furthermore, the correlation between tree height and DBH and knee roots in the middle water table was analyzed. Our results showed that the diameter at breast height and tree height of *T. ascendens* were positively and significantly correlated with the number and size of knee roots (Table 2). The number and the weight of underground roots were significantly correlated with the mean height and surface area of knee roots.

**TABLE 2:** Correlation coefficient between number and size of knee roots and DBH and tree height of *Taxodium ascendens* at Zhaoguan Forest Farm.

	Number of knee roots	Mean height of knee roots	Mean diameter of knee roots	Surface area of knee roots
Tree height	0.45*	0.46*	0.52*	0.52*
Tree DBH	0.66**	0.61**	0.65**	0.59**
Number of underground roots		0.87**	0.48	0.86**
Weight of underground roots		0.93**	0.53	0.95**

*DBH, diameter at breast height.*

*P = 0.05 significance; **P = 0.01 significance.

### 1-Aminocyclopropane-1-Carboxylic Acid Content and 1-Aminocyclopropane-1-Carboxylic Acid Synthetase and 1-Aminocyclopropane-1-Carboxylic Acid Oxidase Enzyme Activities of the Roots

**FIGURE 5:**
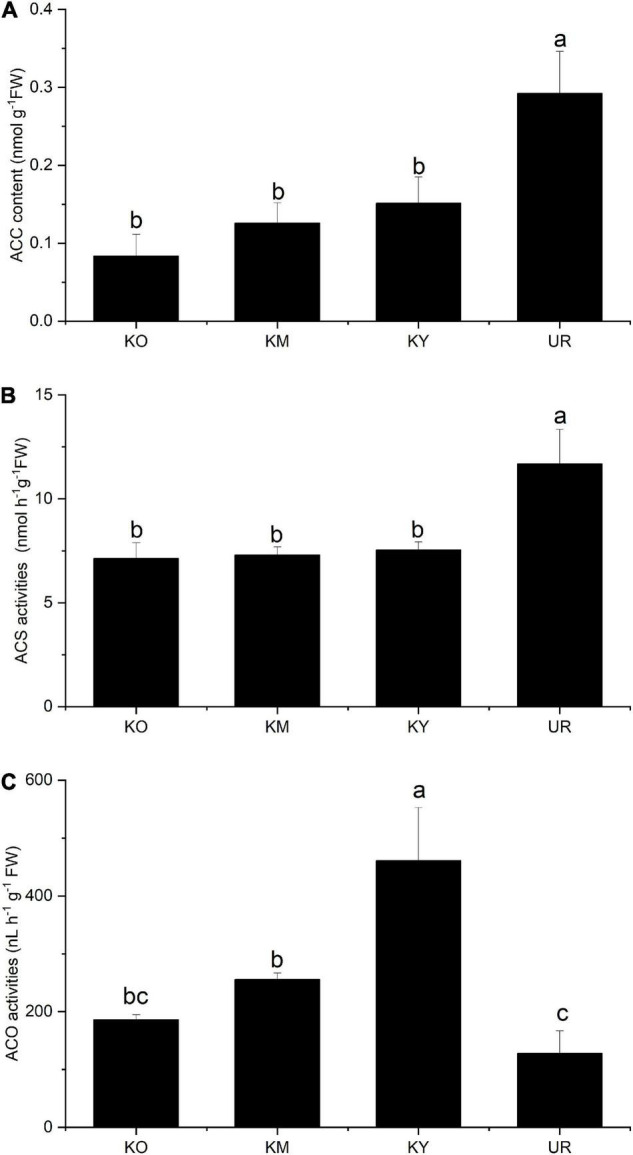
ACC content **(A)** and ACS **(B)** and ACO **(C)** activities in different roots of *Taxodium ascendens.* KO, KM, and KY refer to the knee roots at different development stages: old-aged stage, middle-aged stage, and young-aged stage, respectively. UR means underground roots. ACS, 1-Aminocyclopropane-1-carboxylic acid synthetase; ACO, 1-Aminocyclopropane-1-carboxylic acid oxidase. Values followed by the same letter(s) are not significantly different at *P* < 0.05, according to Duncan’s multiple range tests. Error bars are SD of the mean; *n* = 3.

The ACC content ranged from 0.08 to 0.29 nmol g^–1^FW among the four stages. There was no significant difference in the ACC content among the knee roots at different development stages (Figure 5A). However, the ACC content was significantly lower in the knee roots than underground roots (*P* < 0.05). There were no significant differences in the ACS activity among the development stages of the knee roots (Figure 5B); underground roots exhibited significantly higher ACS activity as compared to knee roots (*P* < 0.05). KY showed significantly higher ACO activity among the development stages of the knee roots (*P* < 0.05); The ACO activity decreased with the growth of knee roots (Figure 5C). There was no significant difference between UR and KO.

### Endogenous Hormone Release Rates

**FIGURE 6:**
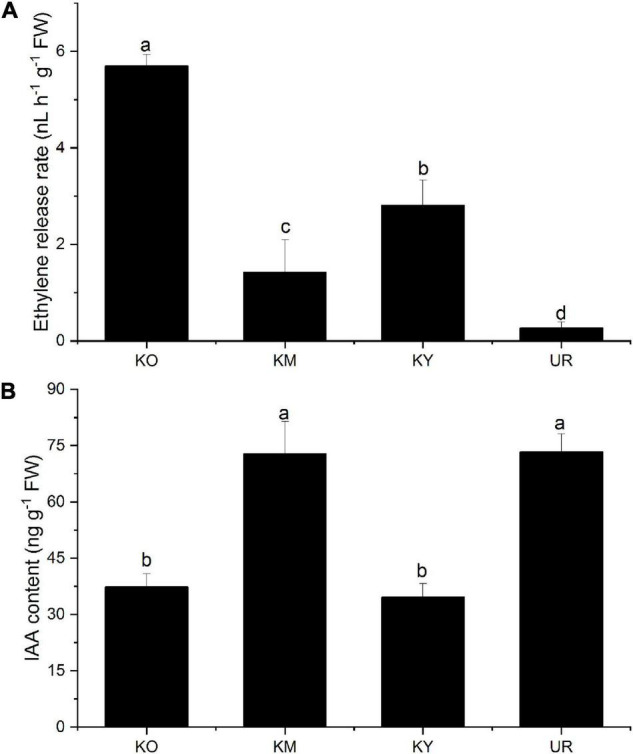
Ethylene release rate **(A)** and IAA content **(B)** from different root types of *Taxodium ascendens.* Values followed by the same letter(s) are not significantly different at *P* < 0.05, according to Duncan’s multiple range tests. Error bars are SD of the mean; *n* = 3.

Different ethylene release rates were observed in the underground roots and knee roots (Figure 6A). The ethylene release rate was significantly higher in the knee roots than in the underground roots (*P* < 0.05). In addition, significant differences were observed among the different development stages of the knee roots (*P* < 0.05). The maximum ethylene release rate from the knee roots was observed in KO, and the minimum, in KM.

The IAA content was in the order of UR > KM > KO > KY (Figure 6B), with no statistically significant differences between UR and KM (*P* > 0.05). There was no significant difference between KO and KY (*P* > 0.05).

### Anatomical Structure of the Knee Roots and Underground Roots

As shown in Table 3, the thickness of the rhytidome increased with the development of the knee roots. The thickness of the rhytidome in the knee roots was significantly greater than that in the underground roots (*P* < 0.05). The radial width of the secondary phloem followed the order of UR < KY < KM < KO. The size of phloem parenchyma increased with the development of the knee roots, the phloem parenchyma of KY was significantly smaller than that of UR (*P* < 0.05). The tangential width of the tracheids also increased with the development of the knee roots. However, the radial width of the earlywood tracheids decreased with the development of the knee roots.

**TABLE 3:** The thickness of the rhytidome, size of the secondary phloem, phloem parenchyma, and earlywood tracheids from the apex of underground roots and knee roots at different development stages.

		KY	KM	KO	UR
Thickness of the rhytidome (mm)		1.53 ± 0.23c	2.51 ± 0.41b	4.07 ± 0.58a	0.24 ± 0.08d
Radial width of the secondary phloem (mm)		0.31 ± 0.03c	0.45 ± 0.03b	1.25 ± 0.03a	0.22 ± 0.02d
Size of the phloem parenchyma	Tangential width (μm)	25.07 ± 2.82b	26.49 ± 5.73ab	29.78 ± 3.54a	28.49 ± 2.93a
	Radial width (μm)	10.72 ± 0.87a	11.08 ± 1.22a	11.14 ± 1.47a	12.75 ± 2.10a
Size of the tracheids	Tangential width (μm)	26.99 ± 2.96b	29.47 ± 7.28b	36.86 ± 5.49a	29.61 ± 5.31b
	Radial width (μm)	37.83 ± 7.19ab	35.39 ± 5.77b	33.34 ± 8.39b	40.37 ± 4.89a

*KO, KM, and KY refer to the knee roots at different development stages: old-aged stage, middle-aged stage, and young-aged stage, respectively. UR means underground roots. Values followed by the same letter(s) are not significantly different at P < 0.05, according to Duncan’s multiple range tests. Data represent mean ± SD values of 9 replicates.*

**FIGURE 7:**
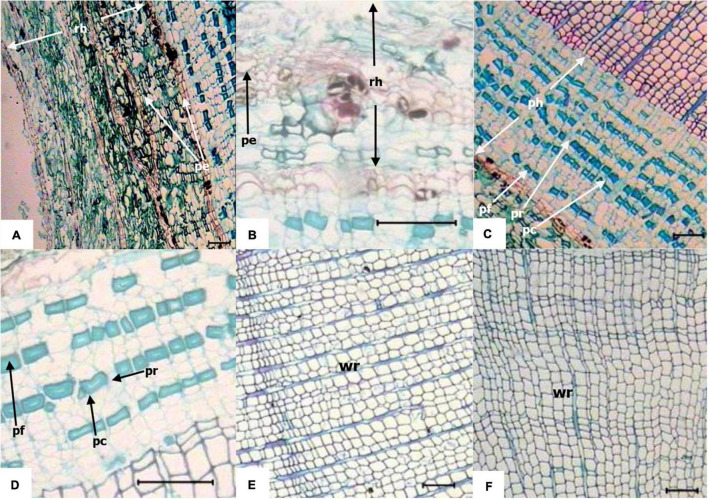
Transverse anatomical structure of knee roots and underground roots. Horizontal line = 100 μm. **(A)** Rhytidome at the apex of the knee roots (middle stage); **(B)** rhytidome of mid-sized underground roots; **(C)** phloem at the apex of the knee roots (middle stage); **(D)** phloem of mid-sized underground roots; **(E)** xylem at the apex of the knee roots (middle stage); **(F)** xylem of mid-sized underground roots; rh, rhytidome; pe, periderm; ph, phloem; pr, phloem ray; pf, phloem fiber; pc, phloem parenchyma cell; wr, wood ray.

The anatomical structures of middle-aged stage knee roots and underground roots were shown in Figure 7. The knee roots had 3–4 layers of rhytidome, which were formed by integrating the periderm and phloem, and some parts of the periderm had branches. The periderm was composed of many layers of cork cells, and the cork layer, cork-forming layer, and inner cork layer were closely overlapped. The phloem, isolated from the periderm on the apex side of the knee roots, was dead, the cells were broken, and the arrangement of cells was loose and porous. The phloem parenchyma cells were close to the cambium and rectangular. The cell wall of the phloem fiber was thickened and showed an increase in lignification. The phloem ray of knee roots was more expanded than that of underground roots. The knee roots were mainly composed of the secondary xylem; in the cross-section, xylem tracheids were arranged in order; the early tracheids were rectangular, square, or polygonal; the late tracheids were smaller than the early tracheids; the cell wall was thicker.

The rhytidome layers of the underground roots were lesser than those of the knee roots (1–2 layers). The phloem cells isolated by periderm were loosely arranged and more orderly than the knee root. The composition and arrangement of secondary phloem were the same as the knee root, but the width of the phloem was smaller than that of the knee roots. The xylem tracheids were arranged in order, and the shape was similar to the knee roots.

## Discussion

**FIGURE 8:**
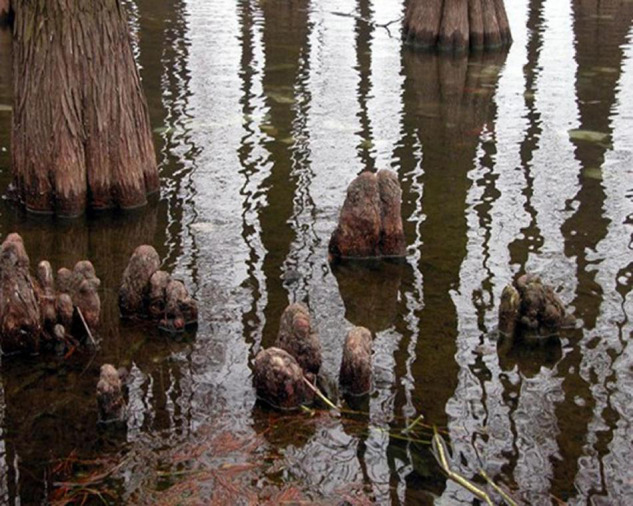
Knee roots of *Taxodium ascendens* in the wetland.

In plants, flood tolerance is related to changes in anatomical and morphological characteristics (Jackson and Colmer, 2005; Hua et al., 2017). The formation of knee roots is a morphological adaptation of *T. ascendens* to environmental stress (Figure 8). In the present study, *T. ascendens* with knee roots had more underground roots and showed better tree growth, suggesting that the knee roots are beneficial for *T. ascendens* under flooding conditions. Our results also indicate that the middle water table is more suitable for the formation and development of knee roots, which is similar to the study of Tang et al. (2008), who reported that *T. ascendens* formed adventitious roots in the high water table and knee roots in the middle water table. The adventitious roots were usually attached to the tree trunk or bark gap, while knee roots were mainly vertical woody outgrowths rising from lateral roots (Rogers, 2021). The low water table was also not conducive to the formation of knee roots. The reason might be the low water table was easier for *T. ascendens* to obtain nutrients and oxygen; there was no need to form the knee roots to resist the flooding stress (Chen et al., 2021; Middleton et al., 2021).

Ethylene can trigger programmed cell death during aerenchyma formation, which is vital in modifying the response of plants to flooding stress (Mignolli et al., 2020). Ethylene synthesis includes two essential processes: catalysis of S-adenosylmethionine to ACC by ACS and catalysis of ACC to ethylene by ACO (Vanderstraeten et al., 2019). ACC can be synthesized under hypoxia conditions in the underground roots; ACS activity is increased in plant roots under flood stress (Dong et al., 1992; Zhou et al., 2001; Williams and Golden, 2002). In our study, the submerged underground roots of *T. ascendens* were exposed to anoxic conditions, which may stimulate the expression of the ACS gene (Van der Straeten et al., 2001; Rieu et al., 2005). Thus the significantly higher ACS activity was observed in the underground roots. However, the formation of knee roots alleviated the anoxic stress of *T. ascendens*, possibly resulting in significantly lower ACS activity in the knee roots. Oxygen is necessary for the process of ACC to ethylene. ACO activity can also be induced by anoxic stress (Vriezen et al., 1999; Bailey-Serres and Voesenek, 2008; Buttò et al., 2020). Thus, in the present study, ACO activity decreased with the development of knee roots, which suggested that the oxygen status was improved by the formation and development of knee roots. When the underground roots of *T. ascendens* were exposed to flooding in the growing season, the activity of ACS was enhanced by anaerobic stress, which led to the highest accumulation of ACC. But the lowest ethylene was observed in underground roots. We believe it was due to the middle water table and the formation of the knee roots. When the water table receded, the upper surface of the underground roots can receive oxygen easily, which contributes to the ethylene synthesis by ACO enzyme; ethylene maybe promotes the formation of knee roots, which resulted in significantly higher ethylene content in the knee roots than the underground roots (Figure 6A). The highest ethylene content was observed in the old-aged stage of the knee roots, which probably contributing to the cell death and lignification in the knee roots formation process (Pesquet and Tuominen, 2011). Besides, ethylene can indirectly regulate gene expression through the signal transduction pathway in plants tissues (Sairam et al., 2008; Zhang et al., 2014). Fan et al. (2018) reported *ThRAP2.3* is one of the critical downstream-response ethylene response factor genes, which can respond to the ethylene signal under flooding stress in a *Taxodium* hybrid “Zhongshanshan 406.” The molecular regulation of ethylene in *T. ascendens* is still unknown, which merits further study.

The phytohormone IAA promotes root development and adventitious root formation (Visser and Voesenek, 2004; Kitomiy et al., 2008). IAA also participates in the development and activities of the cambium (Uggla et al., 1998; Bhalerao and Fischer, 2014). Our study suggests that, with the development of the knee roots, the IAA content first increased, then decreased; IAA content was lower in the knee roots at the young-aged and old-aged stages and higher in those at the middle-aged stage. High IAA content is beneficial for cambium cell enlargement, expansion, and division (Bhalerao and Fischer, 2016), whereas low IAA content is beneficial for secondary cell-wall deposition and lignification (Fajstavr et al., 2018). Our results were similar to the conclusion of Cui et al. (1999, 2000), who reported that endogenous IAA concentration in the cambium of *Broussonetia papyrifera* increased when the cambium formed the immature phloem and xylem; IAA concentration decreased when the immature vascular cells differentiated toward maturation. This result indicates IAA concentrations are different in the division stage of cambium cells and the differentiation stage of cambium derivative cells.

Under flood conditions, anoxia stimulates ethylene production and accumulation in roots, which contributed to the programmed cell death in the cortex tissue (He et al., 1996; Drew et al., 2000). In our study, the periderm cells at the apex of the knee roots were dead, arranged loosely, and had a large number of intercellular spaces, which is conducive to gas exchange between the knee roots and air. Because the knee roots were exposed to air to resist the adverse effects of external environmental factors, the periderm of the knee roots was thicker than that of the underground roots (Table 3). The phloem rays at the apex of the knee roots expanded when compared with the underground roots, possibly due to the ethylene accumulation (Pallardy, 2011).

**FIGURE 9:**
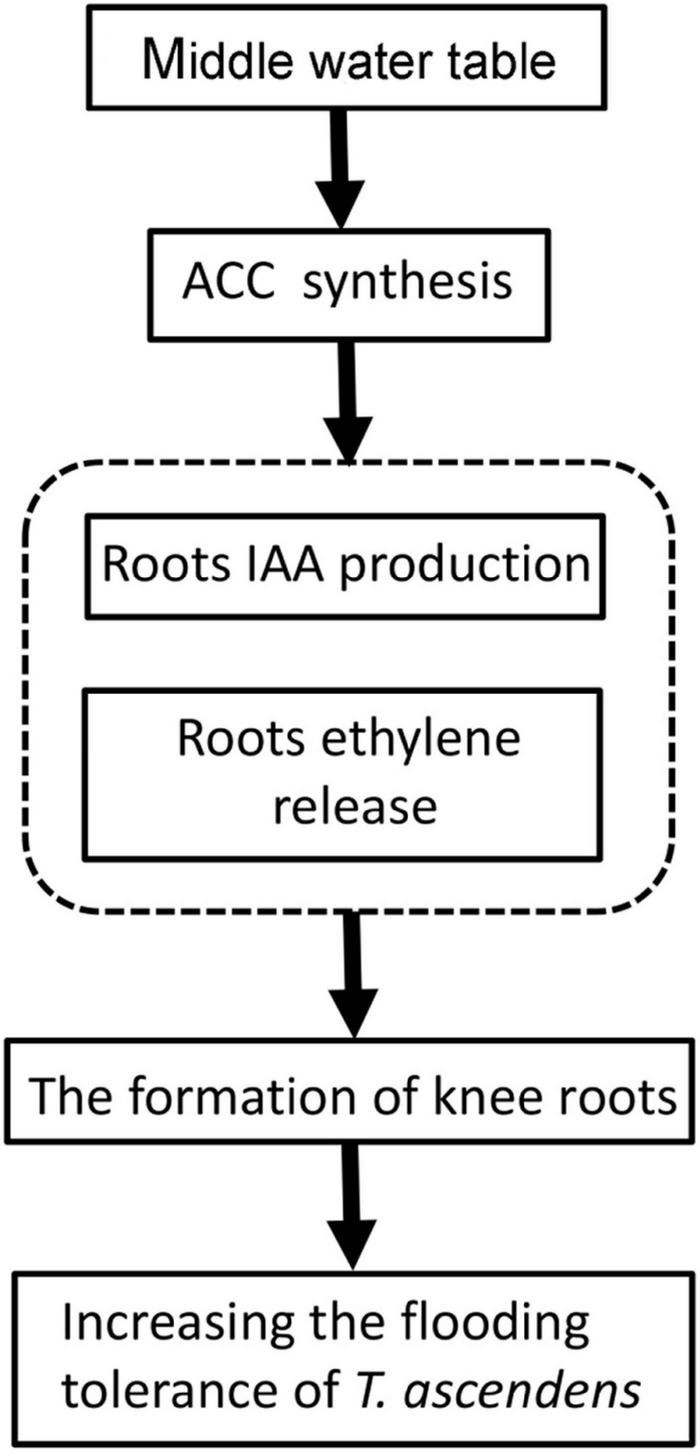
A schematic summary figure showing the adaption mechanism of *Taxodium ascendens* to flooding stress.

In conclusion, consistent with our hypothesis, the flooding resistance and growth condition of *T. ascendens* are related to the formation of knee roots and enhancement of air permeability. Our study also suggested middle water table was conducive to the formation of the knee roots compared with the high water table and low water table. The ethylene and IAA may affect the formation of knee roots (Figure 9). In this study, we realized that the adaptation mechanism of *T. ascendens* to flooding stress is preliminary. The underlying formation mechanism of knee roots is still unclear. We recommend further studies of the formation mechanism of knee roots should be explored to understand the mechanism of flooding resistance.

## Conclusion

In the present study, the middle water table significantly enhanced the formation and distribution of knee roots in *T. ascendens*. Furthermore, the knee roots were beneficial for the development of *T. ascendens*. The underground roots showed significantly higher ACC content and ACS activities than knee roots. Besides, the ethylene release rate was significantly higher in the knee roots than in the underground roots. The highest IAA content was observed in the middle-aged knee roots. The thickness of the rhytidome increased with the development of the knee roots. Cells of the periderm at the apex of the knee roots were dead, arranged loosely, and had a large number of intercellular spaces that improved internal gas diffusion. In conclusion, the middle water table induced IAA and ethylene production, which changed the morphology and anatomy of *T. ascendens* roots. The formation of knee roots can improve roots ventilation and growth of *T. ascendens*.

## Data Availability Statement

The original contributions presented in the study are included in the article/supplementary material, further inquiries can be directed to the corresponding author.

## Author Contributions

LW and LT designed the experiment. ZQ wrote the draft. LT and ZQ revised the manuscript. LW and ZQ carried out the field and laboratory experiments. All authors have contributed to the corrections of the manuscript and approve it for submission.

## Conflict of Interest

The authors declare that the research was conducted in the absence of any commercial or financial relationships that could be construed as a potential conflict of interest.

## Publisher’s Note

All claims expressed in this article are solely those of the authors and do not necessarily represent those of their affiliated organizations, or those of the publisher, the editors and the reviewers. Any product that may be evaluated in this article, or claim that may be made by its manufacturer, is not guaranteed or endorsed by the publisher.
